# Identification of rare levels of methylated tumor DNA fragments using an optimized bias based pre-amplification-digital droplet PCR (OBBPA-ddPCR)

**DOI:** 10.18632/oncotarget.26315

**Published:** 2018-11-16

**Authors:** Mario Menschikowski, Carsten Jandeck, Markus Friedemann, Brit Nacke, Saskia Hantsche, Oliver Tiebel, Olga Sukocheva, Albert Hagelgans

**Affiliations:** ^1^ Institute of Clinical Chemistry and Laboratory Medicine, Carl Gustav Carus University Hospital, Technical University of Dresden, Dresden, Germany; ^2^ School of Health Sciences, Flinders University of South Australia, Adelaide, Australia

**Keywords:** liquid biopsy, free circulating DNA (fc-DNA), methylated DNA, tumor DNA, digital PCR

## Abstract

**Background:**

The analysis of aberrant DNA methylations is used for the diagnosis of cancer as significant changes in the gene methylation pattern are often detected during early carcinogenesis. In this study, we evaluated the performance of a two-step method that combines pre-amplification with ddPCR technique.

**Results:**

By using ddPCR, the dependence of amplification efficiency for methylated and unmethylated DNA fragments on the relevant MgCl_2_ concentration and the annealing temperature was established in addition to the primer design. We found that the efficiency can be adjusted toward methylated sequences by using primers covering one to four CpG sites under appropriately selected MgCl_2_ concentration and annealing temperature. Applying a PCR bias between 85% and 95%, five copies of methylated tumor DNA fragments were detected against a background of 700,000 copies of unmethylated DNA fragments with a high signal-to-noise ratio. The analysis of serum samples from patients with prostate cancer showed a significantly improved performance of the new method in comparison with the MS-HRM technique, ddPCR alone, or ddPCR in combination with an unbiased pre-amplification using methylation-independent primers.

**Conclusions:**

We define this method as an optimized bias-based pre-amplification-digital droplet PCR (OBBPA-ddPCR) technique. This novel method is recommended for the early detection of cancer-specific DNA methylation biomarkers in the form of a liquid biopsy.

## INTRODUCTION

Malignant disease prognosis strongly depends on the stage at which the disease is diagnosed and the presence of metastases. The detection of malignancy at the early stage increases the chances of a positive prognosis. Thus, there is a need to find early cancer biomarkers as well as diagnostic methods allowing their detection with high sensitivity and specificity. Epigenetic mechanisms such as DNA methylation play an important role in many physiological and pathophysiological processes including carcinogenesis [1–5]. Based on the observation that changes in methylation pattern occur early in carcinogenesis, analyses of aberrant DNA methylation patterns have attracted considerable interest as a potential early and reliable biomarker for the initiation of cancer onset and for the further monitoring of disease progression after treatment [1–5].

Several different methods were described for the identification of changed methylation patterns of selected DNA fragments in blood, urine and other human body fluids (which can be collected as liquid biopsy), smears, and histological specimens. The suggested methods, with the exception of the methylation-sensitive restriction enzyme analysis (MSRE-PCR), use bisulfite-modified genomic DNA. The analytical sensitivities of these methods for DNA methylation vary greatly. Direct bisulfite sequencing according to Sanger, has a sensitivity of 10-20%, while pyrosequencing and MALDI-TOF-mass spectrometry-based methods reached a sensitivity of 5% [6, 7]. Methylation-specific PCR (MSP), MethyLight, SMART-MSP (Sensitive Melting Analysis after Real Time-Methylation Specific PCR), MS-HRM (methylation-sensitive high-resolution melting), and methyl-BEAMing (beads, emulsion, amplification, and magnetics) demonstrated analytical sensitivities between 0.1% and 1.0% [8–12]. A combination of MethyLight and digital PCR was first described by Weisenberger et al. [13] and an analytical sensitivity of 0.032% was achieved using this combination [14].

One problem with current PCR-based methods concerns the overestimation of methylation. The mistake is caused by poor bisulfite conversion and/or mispriming, especially with methylation-specific primers (MSP) [9, 11, 15–19]. A lower frequency of false-positive data was described using methylation-independent primers (MIP). However, the sensitivity of the methylation analysis was shown to be lower with the use of MIP [6]. Another problem with PCR-based methods designed to analyze DNA methylation level is associated with varying amplification efficiency (PCR bias) of unmethylated and methylated DNA sequences [20–25]. To overcome the observed preferred amplification of unmethylated in comparison to methylated sequences, primers were proposed to include one or two CpG sites as far from the 3′end as possible. Optimization of the annealing temperature was also recommended [7, 23, 24, 26, 27].

Compared to real-time PCR, the digital PCR technique has the advantage that each DNA molecule in its own partition is amplified into methylated and unmethylated DNA strings without competition for primers. Additionally, the PCR bias does not significantly influence the ddPCR data [28]. Additional problems associated with liquid biopsy are the low total amount of cfDNA obtained from blood samples and the relatively low frequency of tumor DNA compared to the excessive background of wild-type DNA, especially in early-stage tumor diseases [29–31]. The first point hinders the execution of biological and technical replicates, as this requires the allocation of the limited sample to separate reactions. The second point is linked to the technical challenge of developing analytical methods that generate a high signal-to-noise ratio.

For this reason, we considered pre-amplifying the cfDNA targets prior to ddPCR. In order to generate signal-to-noise ratio in the ddPCR, allowing for a sufficient distinction between true positive signals and background noise, we first analyzed the influence of the primer design in conjunction with Mg2+ levels and the annealing temperature on the resulting PCR bias. For this purpose, ddPCR offered the advantage of calculating absolute values of DNA copies without the use of calibrators [32, 33].

As gene target, we analysed a specific *PLA2R1* sequence that we identified to be hypermethylated in leukaemia and prostate cancer cells [34, 35]. The phospholipase A_2_ receptor of type M (PLA2R1) regulates several cancer-limiting reactions, including activation of apoptosis, senescence, and inhibition of cell transformation [36–38]. According to our results, we tested and described a new technique for the identification of minute amounts of tumor DNA against a high background of wild-type DNA with high analytical sensitivity (high signal-to-noise ratio) and specificity (low level of false-positive signals). This technique as a proof-of-principle study was verified using blood samples from healthy individuals and patients with prostate cancer for the first time and confirmed the method effectiveness.

## RESULTS

### The impact of primer design, MgCl_2_ concentration and annealing temperature on the PCR bias after pre-amplification using ddPCR

**Figure 1:**
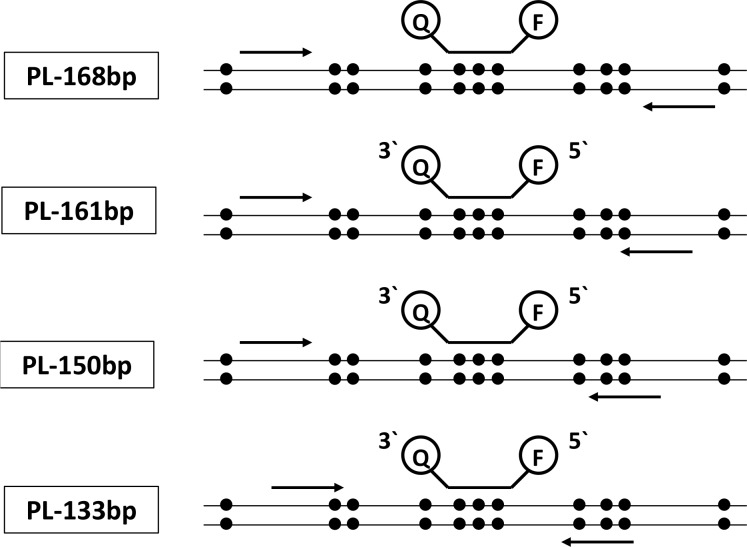
Schematic illustration of the primer and probe localizations related to the presence of CpG sites analyzed in the study, applied in ddPCR alone and in combination with a pre-amplification step Probes were labelled with FAM for methylated and HEX for unmethylated DNA at the 5′-end and Black Hole Quencer 1 at the 3′-end.

**Figure 2:**
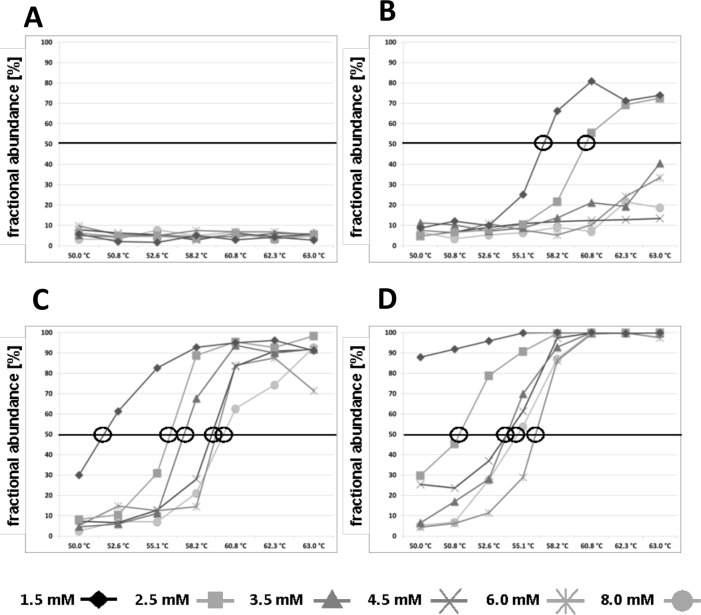
Fractional abundances [%] of methylated DNA fragments of the *PLA2R1* gene in relation to the whole analysed DNA (methylated and unmethylated fractions) after 15 cycles of pre-amplification in dependence of MgCl_2_ concentration and annealing temperature are shown Bisulfite modified standard DNA were applied as templates for pre-amplification and contained 150 copies of methylated and 150 unmethylated *PLA2R1* DNA fragments resulting in an initial fractional abundance of 50%. After pre-amplification, samples were processed on ddPCR. Data were obtained using PL-168 bp primer pair **(A)**, PL-161 bp primers **(B)**, PL-150 bp primers **(C)**, and PL-133 bp primers **(D)**. Circles label the points at which an unbiased pre-amplification was observed. Results are representative data from three independent experiments.

After 15 cycles of PCR pre-amplification, the resulting amounts of DNA copies were determined with ddPCR after 150 copies of methylated and 150 copies of unmethylated DNA were submitted in pre-amplification. Using the MIP primer pair PL-168bp (Figure 1), a preferred amplification of unmethylated DNA fragments compared to methylated DNA fragments was observed, resulting in a fractional frequency from 50.0% to 4.8%±1.6% (Figure 2A and Supplementary Figure 1A). The preferred amplification of unmethylated sequences was unchanged in the temperature range of 50.0°C to 63.0°C and the MgCl_2_ concentration range of 1.5 mM to 8.0 mM.

Using the PL-161 bp primer pair covering a CpG site (Figure 1), a distinct PCR bias towards methylated DNA was achieved in the presence of 1.5 and 2.5 mM MgCl_2_ and annealing temperatures above 55.1°C and 58.2°C, respectively, compared to using MIP (Figure 2B). Above 62.3°C, the fractionated frequency was 70% on average for both MgCl_2_ concentrations. Overall, using the primer pair PL-161 bp showed a lower amplification efficiency for both DNA sequences, which is reflected in the number of positive FAM and HEX events (Supplementary Figure 1B) compared to those observed with the primer pair PL-168bp (Supplementary Figure 1A).

With the primer pair PL-150 bp, which covers two CpG sites, a significant PCR bias towards methylated DNA was observed over the entire temperature range analyzed. This bias was strongly dependent on the concentration of MgCl_2_. The annealing temperature, which was required for a final fractional abundance of more than 50%, rose continuously from 1.5 mM to 8.0 mM as the MgCl_2_ concentration increased. The final fractional abundance reached over 90% (Figure 2C and Supplementary Figure 1C).

As expected, the use of PL-133bp primer pair, which covers four CpG sites with a site near the 3′end of the reverse primer (Figure 1), led to the strongest PCR bias depending on the MgCl_2_ concentration over the entire temperature range of 50.0°C - 63.0°C (Figure 2D). The PCR bias at the annealing temperature of 50.0°C with 1.5 mM MgCl_2_ averaged about 90% and a PCR bias of 100% was achieved at the same MgCl_2_ concentration at annealing temperatures above 55.1°C (Figure 2D).

Conversely, the preferred amplification of unmethylated DNA compared to methylated DNA with primer pair PL-133bp, originally constructed as MSP, was achieved by lowering the annealing temperature and increasing the MgCl_2_ concentration. When the annealing temperature was below 52.6°C and the MgCl_2_ concentration was higher than 3.5 mM, the unmethylated DNA was amplified significantly more than the methylated DNA, resulting in fractional frequencies of <50%. Furthermore, unbiased pre-amplification with the MSP pair was possible, e.g. by using 2.5 mM MgCl_2_ concentration at an annealing temperature slightly above 50.8°C (Figure 2D and Supplementary Figure 1D).

### Analytical sensitivity for rare methylated *PLA2R1*-DNA fragments against a high background of unmethylated DNA fragments dependent on primer design, annealing temperature, and MgCl_2_ concentration

**Figure 3:**
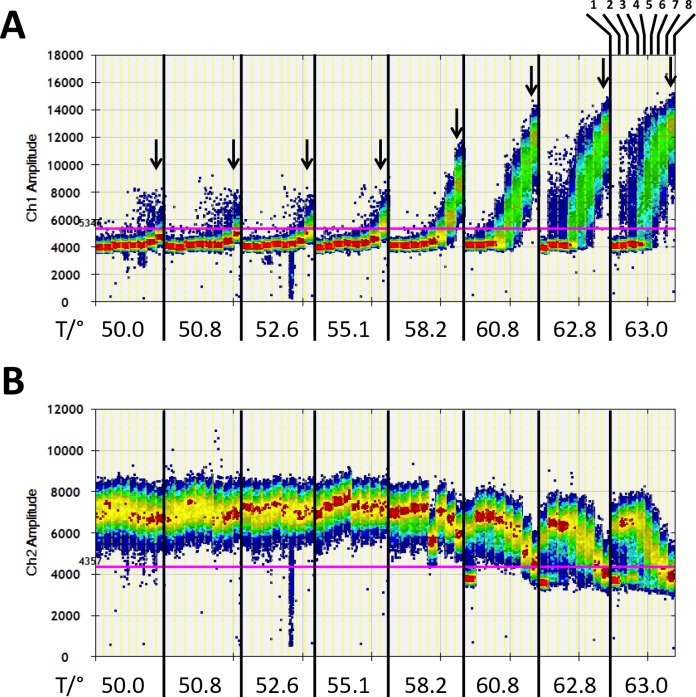
Digital quantification of fluorescence signals in the samples with no or rare number of methylated DNA fragments against a background of 25,000 unmethylated DNA fragments of the *PLA2R1* gene after pre-amplification with 2.5 mM MgCl_2_ for 15 cycles using PL-161 bp primer pair in dependence on annealing temperature **(A)** FAM-signals (Ch1 amplitude, positive for methylated DNA fragments) were registered in samples without (1) or with 5 (2), 9 (3), 94 (4), 375 (5), 750 (6), 1.500 (7), and 3.000 (8) copies of methylated DNA fragments (as shown at the top on the right side). The range of annealing temperature was from 50.0°C to 63.0°C. Arrows show the FAM signal amplitudes of samples with 3,000 copies of methylated DNA dependent on annealing temperature. **(B)** HEX signals (Ch2 amplitude, positive for unmethylated DNA fragments) were registered in the same samples as described in (A). Fluorescence signals are shown as heat map. The used thresholds are shown as red line. Results are representative data from three independent experiments.

Using a first set of DNA control samples consisting of 0, 5, 9, 18, 94, 376, 750, and 3,000 copies of methylated DNA with a background of 25,000 copies of unmethylated DNA fragments, the observed amplitude of FAM positive signals mimicked PCR bias as a function of annealing temperature and 2.5 mM MgCl_2_ concentration in the presence of PL-161bp primer pair (Figure 3). The effect led to a maximum FAM signal amplitude for rare methylated DNA fragments at temperatures above 60.8°C. In the temperature range of 62.8°C-63.0°C, five copies of methylated DNA were already clearly visible against a background of 25,000 unmethylated DNA. High analytical sensitivity was also observed for up to five copies of methylated DNA with the primer pairs PL-150 bp and PL-133 bp at 63.0°C and 2.5 mM and 8.0 mM MgCl_2_, respectively; and at 55.1°C with 1.5 mM and 6.0 mM MgCl_2_, respectively (Supplementary Figure 2). The FAM signal amplitudes in ddPCR with the primer pair PL-168bp were low in comparison. The number of methylated DNA for 94, 375, 750, and 3,000 copies was detectable at 55.1°C with 2.5 mM or 8.0 mM MgCl_2_ and at 63.0°C with 2.5 mM MgCl_2_. However, the detection of the lower amounts of 5, 9, and 18 copies of methylated DNA against the background of 25,000 copies of unmethylated DNA failed with the PL-168bp primer pair (Supplementary Figure 2).

Furthermore, we tested whether the same rare amounts of methylated DNA can be identified with further increased backgrounds of unmethylated DNA using different primer pairs and pre-amplification conditions. To this end, we examined a second set of DNA control samples consisting of 0, 5, 10, 20, and 3,000 copies of methylated DNA with backgrounds of 70,000 to 700,000 copies of unmethylated DNA fragments. First, we analyzed these DNA control samples using ddPCR without pre-amplification. With a background of 70,000 copies of unmethylated *PLA2R1* fragments and 3,000 copies of methylated DNA, there were still enough empty droplets (negative for FAM and HEX signals) with the PL-168bp (MIP) primer pair in the ddPCR (Supplementary Figure 3). According to an average of 13,500 accepted droplets, the copy per droplet (CPD) values for these samples averaged 5.2 and the numbers of 0, 5.4, 11.6, 28.0, and 2,780 copies of methylated DNA were well in line with the 0, 5, 10, 20, and 3,000 copies of methylated DNA used (Supplementary Table 1). After increasing the number of copies of unmethylated DNA to 175,000, 350,000, and 700,000, the recovery of methylated DNA decreased significantly from 2,780 (100%) to 2,018 (73%), 594 (21%), and 120 (4%), respectively. While background DNA increased, analytical sensitivity to the rare methylated DNA decreased continuously, resulting in final detection of only two of the 20 copies presented against a background of 700,000 unmethylated DNA (Supplementary Table 1).

In contrast to the MIP (PL-168bp primer pair), all 3,000 methylated DNA fragments were detectable with the MSP (PL-133bp primer pair, Supplementary Figure 4 and Supplementary Table 2) despite variable backgrounds of 70,000, 175,000, 350,000, or 700,0000 copies of unmethylated DNA fragments. However, rare copies of methylated DNA were also insufficiently detected with this primer pair if the background of unmethylated DNA was increased. In addition, sporadically false-positive FAM signals occurred in samples without methylated DNA when the number of unmethylated DNA increased from 70,000 to 175,000 and 350,000 copies (Supplementary Figure 4), which was not the case when MIP was used (PL-168bp primer pair, Supplementary Figure 3).

Compared to ddPCR alone, we analyzed the same DNA control samples using a pre-amplification step before ddPCR. It was found that 20 copies of methylated DNA were easily detectable against an increasing background of 70,000, 175,000, and 700,000 copies of unmethylated DNA fragments at an annealing temperature of 63.0°C and a concentration of 2.5 mM MgCl_2_ with both PL-161bp and the PL-150bp primer pairs (Supplementary Figure 5). This detection sensitivity was not achieved with primer pair PL-168bp (Supplementary Figure 5, I-III). While the amplitude of the FAM signals decreased continuously using the primer pair PL-161bp when the background of unmethylated DNA increased (Supplementary Figure 5, IV-VI), this did not happen with primer pair PL-150bp. Additionally, in samples with 20 or less methylated DNA fragments and increased backgrounds of 70,000, 175,000, and 700,000 unmethylated DNA copies, comparable amplitudes of FAM signals were obtained with the PL-150bp pair (Supplementary Figure 5, VII-IX). This recovery rate resulted in an analytical sensitivity of 0.0007%.

**Figure 4:**
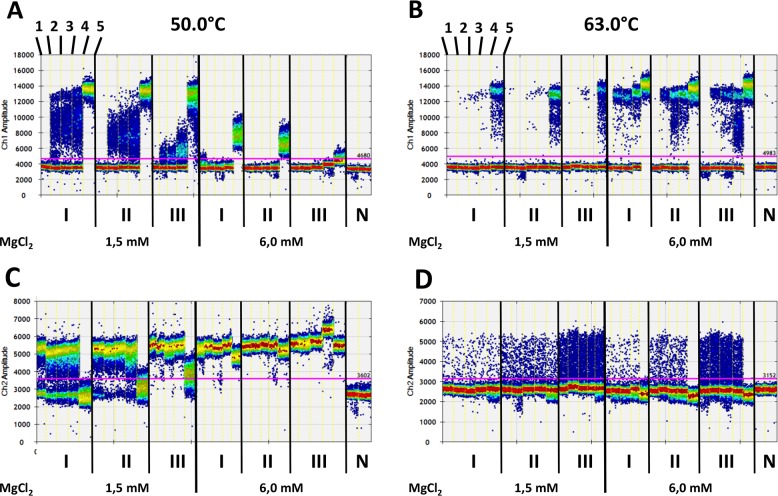
Digital quantification of fluorescence signals in standard samples without (1) or with 5 (2), 10 (3), 20 (4), and 3.000 (5) copies of methylated DNA fragments (as shown at the top on the left side in A and B) against an increasing background of unmethylated DNA fragments (70,000 [I], 175,000 [II], and 700,000 [III] copies) of the *PLA2R1* gene using 15 cycles of pre-amplification with PL-133 bp primer pair **(A)** FAM- (Ch1 amplitude, positive for methylated DNA fragments) and **(C)** HEX-signals (Ch2 amplitude, positive for unmethylated DNA fragments) after pre-amplification with 1.5 mM (left) and 6.0 mM (right) MgCl_2_ concentrations and annealing temperature of 50.0 °C. **(B)** FAM- and **(D)** HEX-signals as described in A and C after pre-amplification with 1.5 mM (left) and 6.0 mM (right) MgCl_2_ concentrations and annealing temperature of 63.0°C. Fluorescence signals are shown as heat map. The used thresholds are shown as red line. Results are representative data from three independent experiments. N; non-template controls.

Using the same set of DNA control samples and the MSP (PL-133 bp) pair, 3,000 copies of methylated DNA were detectable at 6.0 mM MgCl_2_ and an annealing temperature of 50.0°C, but the 5-20 copies of methylated DNA were not (Figure 4A). However, if the MgCl_2_ concentration was reduced from 6.0 mM to 1.5 mM at the same temperature, 5, 10, and 20 copies of methylated DNA could be clearly detected after 15 pre-amplification cycles (Figure 4A). The increase of the annealing temperature from 50.0°C to 63.0°C and a constant MgCl_2_ concentration of 1.5 mM led to a clear detection of 5, 10, 20, and 3,000 copies of methylated DNA against a background of 70,000 unmethylated DNA after 15 cycles of pre-amplification (Figure 4B). However, with a background of 700,000 copies of unmethylated DNA, 5 copies of methylated DNA were not detectable. The increase of the MgCl_2_ concentration from 1.5 mM to 6.0 mM at a constant annealing temperature of 63.0°C resulted in a significantly improved signal-to-noise ratio combined with an increased analytical sensitivity of ddPCR. The samples with only 5 copies of methylated DNA also differed significantly from those without methylated DNA itself with a background of up to 700,000 copies of unmethylated DNA (Figure 4B). This recovery rate again resulted in an analytical sensitivity of 0.0007%. Interestingly, 3,000 copies of methylated DNA were sufficient to completely suppress the generation of HEX positive signals after 15 cycles of pre-amplification in the samples with 70,000, 175,000, and even 700,000 copies of unmethylated DNA, which was not the case when using 1.5 mM instead of 6.0 mM MgCl_2_ concentration at 63.0°C (Figure 4B).

### Detection of methylated *PLA2R1* tumor DNA in serum samples of prostate cancer patients using MS-HRM, ddPCR on its own, and ddPCR after pre-amplification

**Figure 5:**
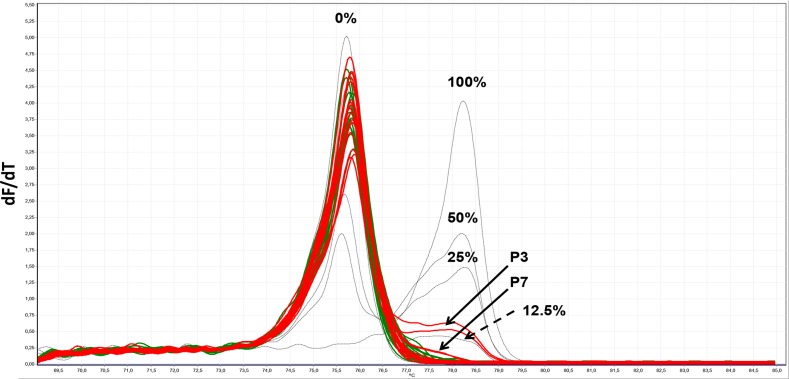
MS-HRM analyses of amplified *PLA2R1* sequences covering nine 5′-CpG-sites [35] after isolation and bisulfite modification of cfDNA from serum of healthy individuals and patients with prostate cancer (as described in Table 2) Melt profiles as negative first derivative of the raw melt pattern (-dF/dT) of 0%, 12.5%, 25.0%, 50.0% and 100% methylated standard DNA samples (dotted lines) and cfDNA from seven pooled serum samples of healthy individuals (in green) and prostate cancer patients (in red) are shown. Arrows show the melt curves found for serum samples of patients P3 and P7 and standard control sample with 12.5% methylation degree. Each sample was analyzed in duplicate by MS-HRM analysis.

**Figure 6:**
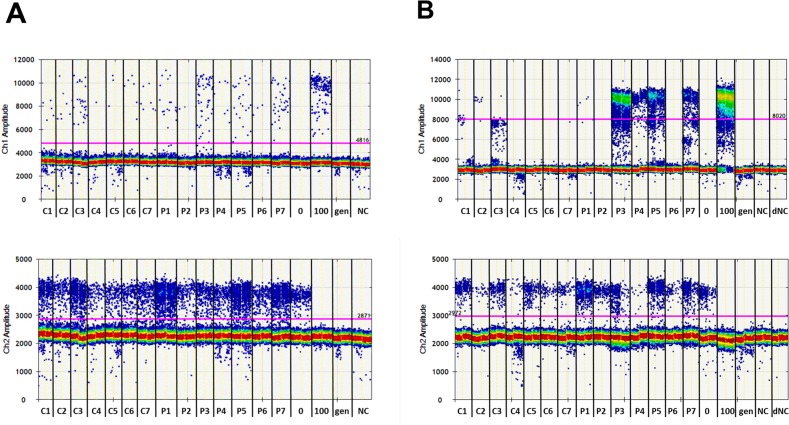
Analyses of the methylation degree of the *PLA2R1* promoter in cfDNA isolated from pooled serum samples of healthy individuals (C1-C7) and patients with prostate cancer (P1-P7) using ddPCR alone **(A)** and the new two-step ddPCR with prior pre-amplification applying primer pair PL-150 bp with 3.5 mM MgCl_2_ concentration and 60.8°C followed by ddPCR **(B)**. ddPCR plots showing FAM and HEX signals for methylated (upper graphs, Ch1 amplitude) and unmethylated (lower graphs, Ch2 amplitude) *PLA2R1* DNA fragments, respectively. Each sample was analyzed in duplicates as shown and are representative of two independent experiments. The used thresholds are shown as red line. 0 and 100, standard DNA with methylation degrees of 0% and 100%; gen, genomic DNA control without bisulfite modification; NC, non-template control for both pre-amplification and ddPCR; dNC, non-template control for ddPCR.

Two of seven pooled serum samples from PCa patients (P3 and P7) were identified as being positive for methylated DNA fragments using MS-HRM, while the serum pools of healthy individuals were negative (Figure 5). Only one of seven patient samples (P3) could be identified as positive with the ddPCR alone (Figure 6A and Supplementary Figure 6A and 6B) or pre-amplification with the MIP (PL-168bp) primer pair and ddPCR (Supplementary Figure 7A-7C). In both cases, the signal-to-noise ratios were low and the P3 sample could only be distinguished from the pools of healthy individuals with a specificity of 100% by using the fractional abundances. In contrast, using the PL-150 bp primer pair (which covers two CpG sites), two further samples from the group of prostate cancer patients, P4 and P5, were identified as positive using pre-amplification prior ddPCR in addition to P3 and P7 samples (Figure 6B, Supplementary Figures 8A and 8B), which were also positive in MS-HRM analysis (Figure 5). A diagnostic specificity of 100% was achieved when a threshold was used to differentiate between healthy controls and patient samples based on FAM signal amplitude. The samples were classified as positive if both PCR replicates provided significant positive signals. The FAM-positive signals that were detected in control sample 3 (C3) are characterized by a significantly lower signal amplitude compared to that of patient samples (Figure 6B). The presence of the FAM-positive signals can be explained by the occurrence of heterogeneous methylated epialleles as recently demonstrated for the *PLA2R1* gene [39], although at a reasonably low level. The results of this proof-of-principle study are summarized in Table 2.

## DISCUSSION

In the present study, a new two-step method based on ddPCR for the detection of rare tumor-specific methylated DNA fragments against a high background of wild-type DNA fragments was established. The method can in principle be applied to all differently methylated DNA sequences and is based on the analysis of the amplification efficiency for methylated and unmethylated sequences depending on primer design, MgCl_2_ concentration, and annealing temperature. According to the data obtained here, which proved the importance of all these components, a PCR bias between 85% and 95% was considered optimal to obtain a strong signal-to-noise ratio in the final ddPCR analysis.

Using the ddPCR alone, it could be shown that when the CPD value of 6 was significantly exceeded in the ddPCR, i.e. when a total of more than 120,000 DNA copies with theoretically 20,000 droplets were applied to the ddPCR, the recovery rate of methylated DNA copies using MIP pairs decreased continuously (Figure 2). A CPD value greater than 6 indicates that the majority of the droplets contain more than one DNA template, resulting in the same bias as with conventional PCR [9, 20–25]. To overcome the limited sensitivity of the ddPCR method after exceeding the critical CPD value, DNA samples can either be distributed to several tubes with 20,000 droplets each or analyzed in a ddPCR system with a significantly higher number of droplets (or partitions when using chip-based digital PCR). However, both approaches would lead to higher up-front investment and consumables costs.

Another alternative is to use primer pairs with a minimum number of CpG sites in their sequences, according to the data described by Clark et al. [40]. This and subsequent studies recommended the inclusion of one or two CpG sites in the primer sequence and that the CpG sites should be located as close as possible to the 5′ end of the primer sequences [9, 22–27]. This suggestion was confirmed in the current study showing that, in comparison to MIP, the inclusion of one to four CpG sites into the primer sequences significantly increased the analytical sensitivity for the rare number of methylated tumor DNA against a high background within the regulated range of MgCl_2_ concentration and annealing temperature.

According to our data with the PL-133 bp primer pair originally designed as MSP, it was shown that the CpG sites in the primer sequences need not be absolutely limited to the 5′end of the primer pair if the MgCl_2_ concentration and annealing temperature are set accordingly. This characteristic makes the design of primers more flexible and can be particularly important for gene sequences that are characterized by a high density of CpG sites. In such cases, the generation of suitable primers with only one or two CpG sites, whose localization should be limited to the 5′ end of the primer, can be partially complicated. Although the highest analytical sensitivity was described to be achieved in methylation analysis with MS-PCR [10], the major drawback of using MSP was the susceptibility to false-positive results leading to the overestimation of methylation degrees in various studies [9, 11, 15–19]. The exclusive amplification of methylated sequences and potentially false-positive signals with MSP can be reduced by adjusting the MgCl_2_ concentration and annealing temperature accordingly. This way, even if only a certain percentage of unmethylated DNA fragments are amplified, they can be used as internal controls for DNA application, which is not possible directly in MS-PCR. On the other hand, it was even possible to amplify unmethylated DNA fragments in preference to methylated DNA fragments with MSP (Figure 2). At a low annealing temperature (50.0-55.1°C) and an MgCl_2_ concentration above 2.5 mM, a fractional frequency below 50% was obtained after pre-amplification. This observation can be important if unmethylated gene sequences are specific for tumors that additionally have a high density of CpG sites and a primer design that does not cover CpG sites is impossible.

Numerous approaches, methods, and recommendations have previously been published regarding high analytical sensitivity for methylated DNA fragments in an environment where there is a large excess of unmethylated wild-type DNA. Those methods included blocking oligonucleotides named HeavyMethyl PCR [41], Headloop PCR [42], and MS-NaME-PCR [43]. The methods measured exclusively methylated fragments that would not allow determination of real methylation degrees in biological samples of cancer patients. Considering the high level of PCR bias described in the current study, the measured methylation degrees also fail to correspond to the actual values. However, an unbiased pre-amplification can be relatively easily performed if it is necessary to find the real degree of the methylation values. To reach an unbiased PCR, an applied primer pair should cover between one to four CpG sites of the gene sequence of interest. Appropriate selection of MgCl_2_ concentration and annealing temperature (labelled by circles in Figure 2) can easily facilitate an unbiased pre-amplification. The resulting methylation values would correspond to the actual methylation degrees and are not changed during the pre-amplification. This may be important in situations where the methylation degree of the targeted gene sequences is used to control the response before and after cancer treatment. A recent study described a method for overcoming the PCR bias via alternative base substitutions at the primer CpG sites [44]. However, the authors used primers matching neither C nor T in the CpG site, with reduced amplification efficiency. Other primers with basic sites were unbiased for some, but not all, analyzed genes [44]. In comparison, our method recommends optimization of MgCl_2_ concentration along with annealing temperature associated with the selective primer design.

As a proof-of-principle study, we applied the new two-step method, in addition to artificial standard DNA controls, on real biological samples such as serum of patients with prostate cancer in comparison to those of healthy individuals. According to our results, the analytical sensitivity of the new technique was significantly higher compared to the current MS-HRM techniques, ddPCR alone (which is identical to MethyLight ddPCR [13]), or ddPCR combined with an unbiased pre-amplification using MIP. In connection with the serum analyses in this study, it is worth mentioning that the quantification of *GSTP1* methylation based on the new two-step method identified the patient samples P1-P5, but not the sample P7 (in which methylated *PLA2R1* DNA fragments were found) as positive. This suggests that a combination of both markers may improve the diagnostic sensitivity of prostate cancer diagnosis (manuscript in preparation). Further studies are needed to analyze the analytical performance of the new technique using larger amounts of patient blood samples.

A disadvantage of the method described is that the procedure cannot be completed within a closed-tube system, as after the pre-amplification step a portion of amplicons must be transferred into the ddPCR equipment. Therefore, an additional precaution is recommended to prevent sample contaminations. A series of negative controls such as non-template controls in the pre-amplification and ddPCR steps should be included. Non-bisulfite modified genomic DNA controls should be also included in every assay.

In conclusion, this study established a new technique consisting of a pre-amplification step followed by ddPCR procedure for the identification of rare tumor DNA against a high background of wild-type DNA. During pre-amplification, DNA target sequences were preferentially, but not exclusively, amplified using a tumor-specific approach. The utility of primer pairs with different levels and positions of included CpG sites has been shown inaugurating a relatively great flexibility in future primer constructions. A prerequisite is the optimization of MgCl_2_ concentration along with annealing temperature, depending on whether methylated or unmethylated gene sequences are targeted. We demonstrated that minute amounts of tumor DNA can be specifically detected against a high background of wild-type DNA in blood serum samples of prostate cancer patients. We suggest naming the two-step technique described here “optimized bias-based pre-amplification-digital droplet PCR” (OBBPA-ddPCR). This new technique should be tested in future studies seeking a way to differentiate between benign prostate hyperplasia, prostatitis, and prostate cancer, especially if serum PSA values were measured between 2.5 to 10.0 ng/ml.

## MATERIALS AND METHODS

### Patient samples

Serum samples from patients with prostate cancer (N=22) and healthy individuals (N=18) were pooled resulting in two groups of seven prostate cancer samples and seven healthy control samples with 5 ml serum, respectively, allowing the isolation of sufficient amounts of cfDNA for subsequent analysis. Prostate cancers were diagnosed in all patients included in this study by routine histopathological examination of the surgically removed glands or biopsy tissue specimen. Amounts of isolated cfDNA from pooled serum samples, averaged PSA values and Gleason scores of included prostate cancer patients are summarized in Table 2. Use of the patient's samples was approved by the Ethics Committee of the University Hospital ‘Carl Gustav Carus’, Dresden, Germany.

### Cell culture

U937 (human hystiocytic lymphoma) cell line was purchased from the German Collection of Microorganisms and Cell Cultures (DSMZ, Braunschweig, Germany). Cells were cultured in a standard cell culture medium RPMI 1640 supplemented with 10% heat-inactivated fetal calf serum (FCS), 2 mM L-glutamine, 100 U/ml penicillin, and 100 μg/ml streptomycin at 37°C in a humidified atmosphere of 5% CO_2_.

### Isolation of cellular genomic DNA and free-circulating DNA from serum samples

Genomic DNA was isolated from U937 leukemic cell line using a Blood & Cell Culture DNA Mini Kit (Qiagen GmbH, Hilden, Germany) following the manufacturer's instructions. Isolation of cfDNA from 5 ml serum samples was performed using the NucleoSnap DNA Plasma Kit (Macherey-Nagel, Düren, Germany) according to manufacturer's instructions and all DNA samples were stored at – 80°C until analysis.

### Preparation of DNA samples with a 50% methylation degree

Standard DNA samples were prepared with the equal amount of unmethylated and methylated control DNA copies (Qiagen) to quantify the PCR bias. The ddPCR technique makes it possible to collect the accurate number of unmethylated and methylated *PLA2R1*-DNA copies. According to the observed data, 10 ng of bisulfite-modified samples of unmethylated and methylated control DNA (Qiagen) contained about 300 and 400 copies of *PLA2R1* fragments respectively, depending on the charge used. During the pre-amplification step, 150 copies of unmethylated and 150 copies of methylated DNA (given 50% methylation degree) were utilized, keeping the copy number below the copy per droplet (CPD) value of 6 in the following ddPCR procedure after 15 cycles of pre-amplification. This value was recommended as the maximum level allowing the exact quantification of copy numbers based on normal Poisson distribution [32].

### Preparation of DNA samples with rare amounts of methylated fragments against a high background of unmethylated DNA

To assess the analytical sensitivity of the method, standard DNA series were prepared which contained rare amounts of methylated DNA against a high background of unmethylated DNA. To receive sufficient unmethylated control DNA, 10 ng unmethylated genomic control DNA (EpiTect PCR Control DNA kit, Qiagen Cat #59655) was amplified with 5′-TACTCTGGGGCAAGGAAGG-3′ as forward and 5′-TTGCAAACCACCTGGATTCT-3′ as reverse primers. The resulting 176 bp-long amplicons contained sequences of all four primer pairs (Table 1) which were utilized in the following analyses of the PCR bias. Thermal cycling conditions consisted of 95°C for 5 min, 40 cycles of 94 °C for 30 s, 60.0 °C for 30 s, and 72.0 °C for 1 min with a final 7 min hold at 72.0 °C. After PCR amplification, products were purified using the MinElute PCR Purification kit (Qiagen, #28004) according to the manufacturer's instructions. The amplicons were finally eluted in 10 μl BE buffer and stored at – 80°C until analysis.

**Table 1:** Applied primer (A) and probe (B) sequences were used to analyze the PCR bias and amplification efficiency, and identify rare amounts of methylated DNA at a high background of unmethylated wild-type DNA

A			
**Target**	**Forward (5′->3′)**	**Reverse (5′->3′)**	**Amplicon length**
*PLA2R1*	GGGGTAAGGAAGGTGGAGAT	ACAAACCACCTAAATTCTAATAAACAC	168 bp
*PLA2R1*	GGGGTAAGGAAGGTGGAGAT	ACCTAAATTCTAATAAACACCGC	161 bp
*PLA2R1*	GGGGTAAGGAAGGTGGAGAT	AATAAACACCGCGAATTTACAAC	150 bp
*PLA2R1*	GGAAGGTGGAGATTACGG	GCGAATTTACAACGAACAAC	133 bp
**B**			
**Target**	**methylated (5′->3′)**		**unmethylated (5′->3′)**
*PLA2R1*	CCCAACTACTCCGCGACGCAA		AACCCAACTACTCCACAACACAAA

To assess the analytical sensitivity's reliance on the primer design, MgCl_2_ concentration, and annealing temperature, two sets of standard DNA samples were prepared and contained at first 25,000 copies of unmethylated *PLA2R1*-DNA along with 0, 5, 9, 18, 94, 375, 750, and 3,000 copies of methylated *PLA2R1*-DNA from U937 cells. In previous studies we identified the *PLA2R1* promoter hypermethylated in U937 leukemic cell lines and in LNCaP prostate cancer cell line [35, 38, 39]. As the *PLA2R1* promoter was completely methylated in every DNA isolation independent on the used passages of U937 cells and the DNA methylation degree of the *PLA2R1* promoter in LNCaP cells varied between passages in a range of 83-95% we decided to use U937 DNA as model target of methylated tumor DNA. A second set of DNA standard samples was prepared with increasing levels of unmethylated DNA containing 70,000, 175,000, 300,000, and 700,000 copies along with 0, 5, 10, 20, and 3,000 copies of methylated DNA.

### Bisulfite modification of isolated DNA

After determination of the DNA content using Quantus photometer and QuantiFluor dsDNA-System Kit (Promega, Mannheim, Germany), 80 ng of the purified DNA fragments from unmethylated genomic control DNA (EpiTect PCR Control DNA kit, Qiagen) after pre-amplification were bisulfite modified using EpiTect Fast DNA Bisulfite Kit (Qiagen GmbH) according to manufacturer's instructions. The resulting bisulfite modified fragments were eluted in 40 μl EB buffer and quantified with 9.72 × 10^8^ copies/μl (3.0 × 10^8^ copies/ng) of unmethylated *PLA2R1* DNA fragments using ddPCR. Correspondingly, DNA fragments isolated from U937 cells and bisulfite modified methylated *PLA2R1* were quantified with 6,500 copies/μl. After determination of the cfDNA content isolated from pooled serum samples ranging between 152 ng and 2,200 ng (Table 2) all the isolated cfDNA were bisulfite modified and the bisulfite-converted samples were eluted in 45 μl EB buffer and stored at – 80°C until analysis.

**Table 2:** Summerized data received from the analysis of pooled serum samples from healthy individuals (C1-C7) and patients with prostate cancer (P1-P7) using methylation-sensitive high-resolution melt (MS-HRM) analysis, digital droplet PCR (ddPCR) alone, unbiased pre-amplification (UBPA)-ddPCR and optimized bias-based pre-amplification (OBBPA)-ddPCR

ID	ng of cfDNA	PSA (ng/ml)	Gleason score	MS-HRM	ddPCR alone <4.7	UBPA-ddPCR <4.0	OBBPA-ddPCR <1.3
C1	640	-	-	-	-	-	-
C2	320	-	-	-	-	-	-
C3	920	-	-	-	-	-	-
C4	152	-	-	-	-	-	-
C5	320	-	-	-	-	-	-
C6	340	-	-	-	-	-	-
C7	400	-	-	-	-	-	-
P1	2200	21,103 ± 6,510	10	-	-	-	-
P2	560	730.0 ± 141.7	10	-	-	-	-
P3	680	636.9 ± 350.8	10	+++	15.5	13.2	97.0
P4	640	85.7 ± 6.9	9 + 10	-	-	-	96.9
P5	1240	25.5 ± 6.3	9	-	-	-	73.4
P6	224	9.3 ± 2.2	7	-	-	-	-
P7	840	8.2 ± 1.7	6 + 7	+	-	-	69.6

Amounts of isolated cell-free DNA (cfDNA) from 5.0 ml pooled serum samples, averaged PSA values of prostate cancer patients, Gleason scores and c*utoff* values together with fractional abundances observed by the different ddPCR methods are shown.

### Methylation specific-high resolution melt (MS-HRM) analyses

MS-HRM analyses were carried out to quantify the extent of methylation in the distinct region −644 bp to −478 bp from the transcription start site (TSS) of the *PLA2R1* gene [35]. These analyses were carried out using Rotor-Gene Q (Qiagen GmbH) and the EpiTect MS-HRM PCR Kit according to manufacturer's instructions. 2 μl of bisulfite modified cfDNA from patient samples P1-P7 and healthy individuals G1-G7 were applied as duplicates and 10 ng bisulfite modified unmethylated (0%) and methylated (100%) standard DNA (Qiagen GmbH) were used as positive controls and non-template and 30 ng genomic DNA without bisulfite modifications as negative controls in each run. PCR was performed in 12.5 μl volumes. The applied methylation-independent primer (MIP) pairs were 5′-GGG GTA AGG AAG GTG GAG AT-3′ and 5′-ACA AAC CAC CTA AAT TCT AAT AAA CAC-3′, generating PCR products of a length of 168bp. The primers were applied at a final concentration of 0.8 μM. The conditions of amplification were as follows: 40 courses at 95 °C for 10 seconds, 58 °C for 30 seconds and 72 °C for 15 sec. Immediately after PCR, products were analyzed by high resolution melt analysis with fluorescence measured during the linear temperature transition from 50-95 °C at 0.01 °C/second.

### Pre-amplification of bisulfite-modified DNA

Temperature gradient was set up using the built-in function of the CFX thermal cycler (Bio-Rad Laboratories GmbH, München, Germany). PCR master mix (10x) with 15 mM MgCl_2_ concentration was diluted to a final concentration of 1.5 mM MgCl_2_ and used for pre-amplification. Additional MgCl_2_ was added to the master mix to reach final concentrations of 2.5, 3.5, 4.5, 6.0, and 8.0 mM MgCl_2_ as indicated.

During the pre-amplification step, bisulfite-modified DNA standard samples, as described below, were applied. The volume of 25 μl of PCR reactions including 0.625 U of HotStarTaqPlus (Qiagen) and 200 μmol/l of dNTPs were used. Primers were applied at a final concentration of 400 nmol/l. The thermal cycling conditions were 95°C for 5 min and 15 cycles of 94°C for 10 s, different annealing temperatures as indicated for 30 s followed by 72°C for 30 s, and a final hold at 4°C.

Four different primer pairs were designed using Primer3Plus software (http://www.bioinformatics.nl/cgi-bin/primer3plus/primer3plus.cgi) and Methprimer software [45], (https://www.urogene.org/methprimer/). PL-168bp primers (as MIP pair) were designed avoiding CpG sites in their sequences (Figure 1 and Table 1). The PL-161 bp primer pair included one CpG site near the 3′-end of the reverse primer, whereas the forward primer was free of CpG sites. The reverse primer of the PL-150 bp primer pair included two CpG sites in the middle of the primer sequence, while the forward primer was free of CpG sites. PL-133 bp primers (as MSP pair) were designed so that the reverse primer included three CpG sites at the 5′-end, and the forward primer contained one CpG site near the 3′-end (Figure 1 and Table 1). All primers were proofed during the developing phase through the confirmatory gel electrophoresis and demonstrated only one band of the expected sizes (data not shown).

Duplicates of 2 μl of bisulfite modified cfDNA from patient samples P1-P7 and healthy individuals G1-G7 were preamplified using primer pairs and amplification conditions as indicated. 10 ng bisulfite modified unmethylated (0%) and methylated (100%) standard DNA (Qiagen GmbH) were used as positive controls and non-template and 30 ng genomic DNA without bisulfite modifications as negative controls in each run.

### Digital droplet PCR of bisulfite-modified DNA

All ddPCR analyses were performed using the QX100 Droplet Digital PCR System according to the manufacturer's instructions (Bio-Rad). Each amplification ddPCR reaction mixture consisted of the 2X ddPCR supermix for probes and primers. Probe sequences were designed using OligoArchitec software from Sigma-Aldrich (Taufkirchen, Germany) and are listed in Table 1B. Probes were synthesized at 5′-end with FAM for methylated sequences, HEX for unmethylated sequences, and at the 3′-end with BHQ-1 as fluorescence quencher. During the ddPCR procedure the same primer pairs were applied as during the pre-amplification step. The optimal annealing temperature for ddPCR, 58.8°C, was established in preliminary experiments using the designed primers and probes for analysis of 0% and 100% methylated standard DNA controls (Qiagen, data not shown).

Primer and probes (Table 1) were used in the ddPCR analysis at final concentrations of 900 nmol/l and 250 nmol/l respectively. Each 20 μl PCR reaction sample containing either 2 μl of bisulfite-modified DNA without pre-amplification or 2 μl DNA templates after pre-amplification was loaded into the Bio-Rad DG8 disposable droplet generation cartridges (Bio-Rad). A volume of 20 μl of droplet generation oil was loaded into adjacent wells. Microfluidic chips were then loaded into a droplet generator (BioRad). Overall, 10.000 to 17.000 accepted droplets were generated per reaction. The water-in-oil droplets were pipette-transferred from the outlet well to a 96-well polypropylene plate. The heat-sealed plate was placed in a T100 Thermal Cycler (BioRad) and amplified for 40 cycles to the endpoint. The thermal cycling conditions included 95°C for 10 min, 40 cycles of 94°C for 30 s, and 58.8°C for 1 min with a final 10 min hold at 98°C. After PCR amplification was complete, the 96-well plate was loaded into a QX100 droplet reader (BioRad). All methylation quantification experiments included no-template controls (NTCs and ddNTCs), which contained all the components of the reaction without DNA templates at the beginning of the pre-amplification step and during the following ddPCR as well as at the beginning of the ddPCR. Additionally, 30 ng genomic DNA (gDNA) was analysed as supplementary negative control in every assay. Data were only analyzed using the QuantaSoft version V1.6.6.0320 (Bio-Rad) when all controls were negative. A blood sample was deemed positive if both ddPCR replicates were positive for methylated *PLA2R1* fragments.

## SUPPLEMENTARY MATERIALS FIGURES AND TABLES






