# Supplementary material for: Identification of rare levels of methylated tumor DNA fragments using an optimized bias based pre-amplification-digital droplet PCR (OBBPA-ddPCR)
Source: Oncotarget. 2018 Nov 16;9(90):36137–50. doi: 10.18632/oncotarget.26315 (PMC6281424; doi:10.18632/oncotarget.26315)
Supplement: Supplementary file 1 [file oncotarget-09-36137-s001.pdf]

Identification of rare levels of methylated tumor DNA fragments using an optimized bias based pre-amplification-digital droplet PCR (OBBPA-ddPCR)

SUPPLEMENTARY MATERIALS

REFERENCE

1. Applications Guide dPCR, Biorad. http://www.bio-rad.com/webroot/web/pdf/lsr/literature/Bulletin_6407.pdf.

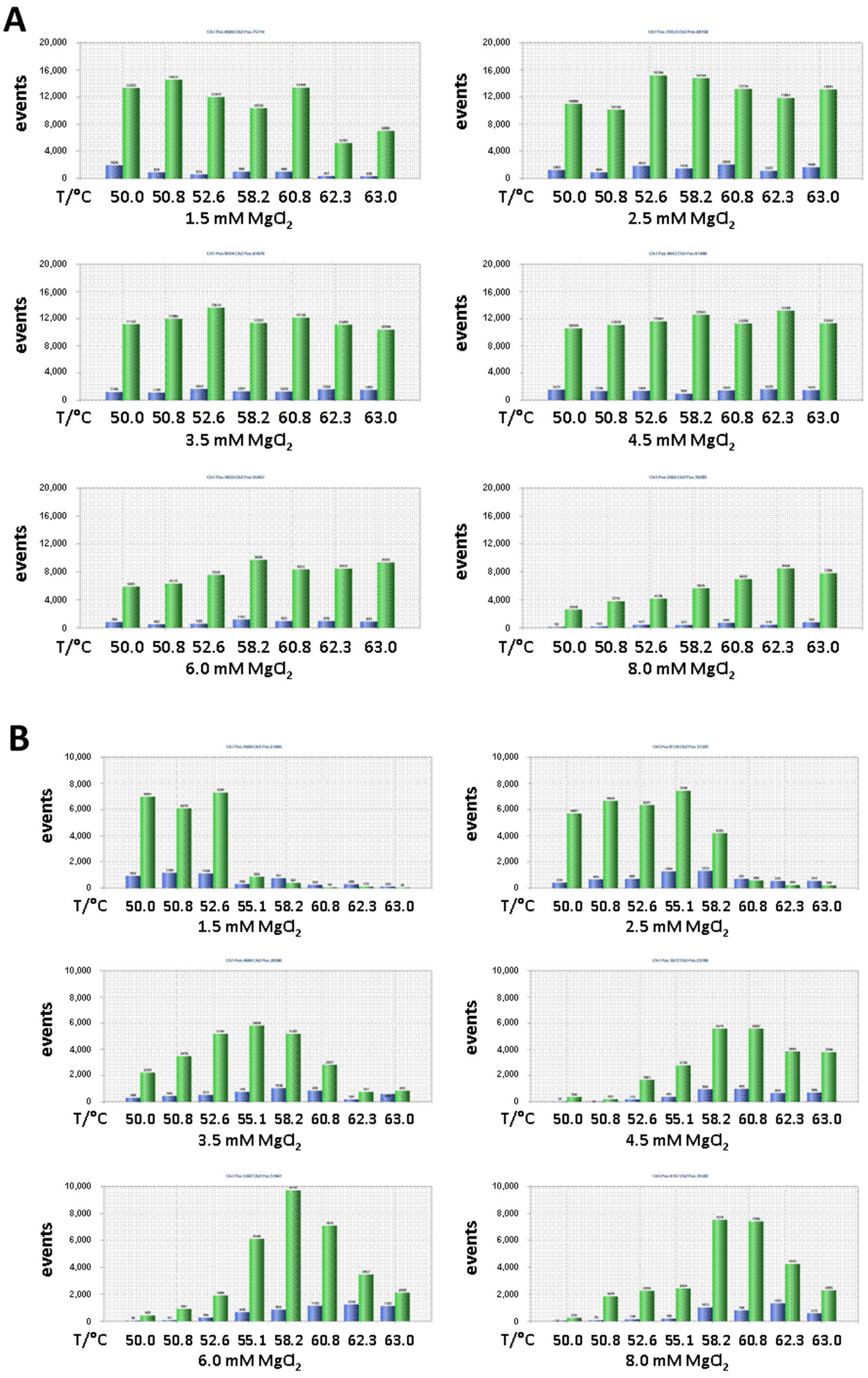

Supplemenary Figure 1: (A-D) Quantification of positive events for methylated (in blue) and unmethylated PLA2R1 DNA fragments (in green) after 15 cycles of pre-amplification in dependence on primer design, $MgCl_2$ concentrations and annealing temperatures after quantification using ddPCR are shown. 150 copies of methylated and 150 copies of unmethylated *PLA2R1* DNA fragments were applied as templates.

C

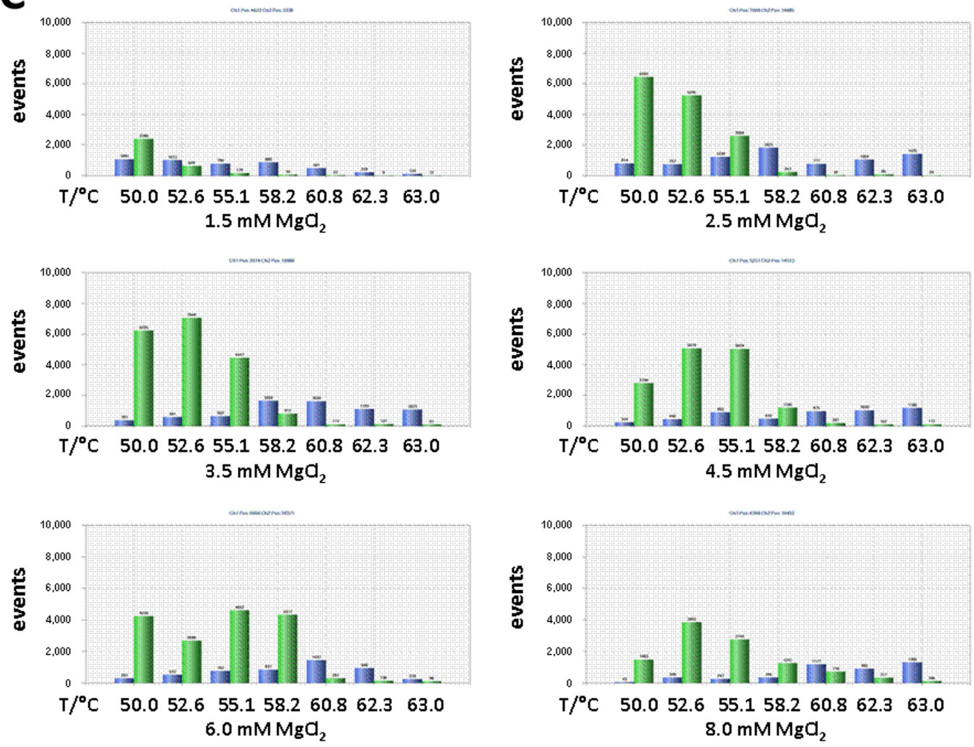

D

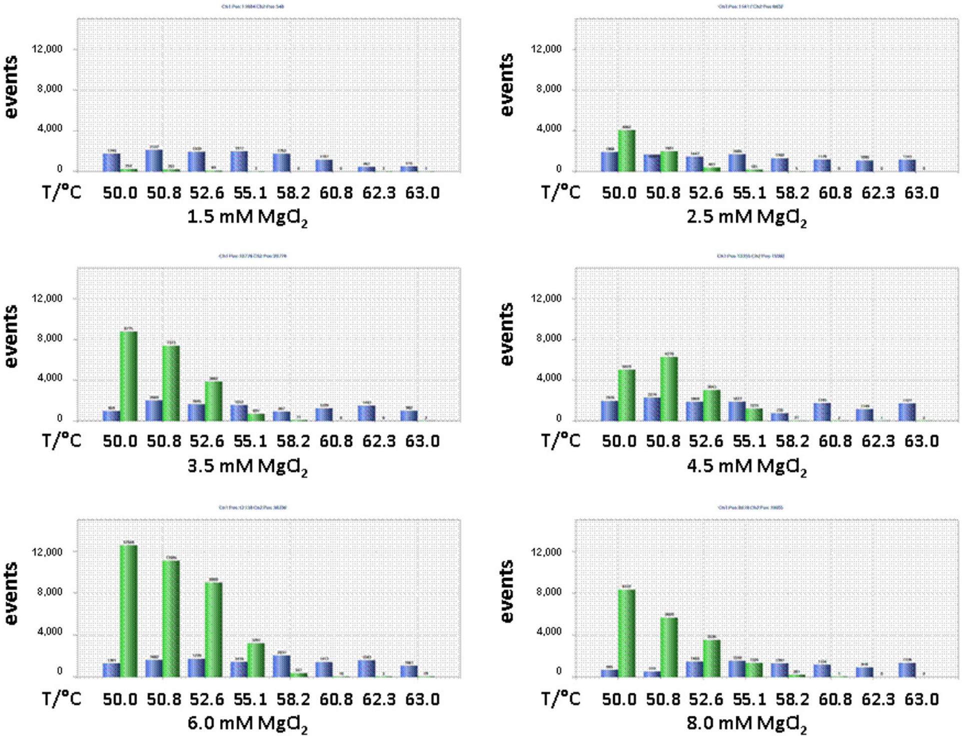

Supplemenary Figure 1: (Continued) Data received using PL-168bp (Supplemenary Figure 1A), PL-161bp (Supplemenary Figure 1B), PL-150bp (Supplemenary Figure 1C), and PL-133bp (Supplemenary Figure 1D) primer pairs in dependence on $MgCl_2$ concentrations (1.5 mM, 2.5 mM, 3.5 mM, 4.5 mM, 6.0 mM and 8.0 mM as indicated at the bottom of the graphics, respectively) and at increasing annealing temperature (T/°C). Results are representative of three independent experiments.

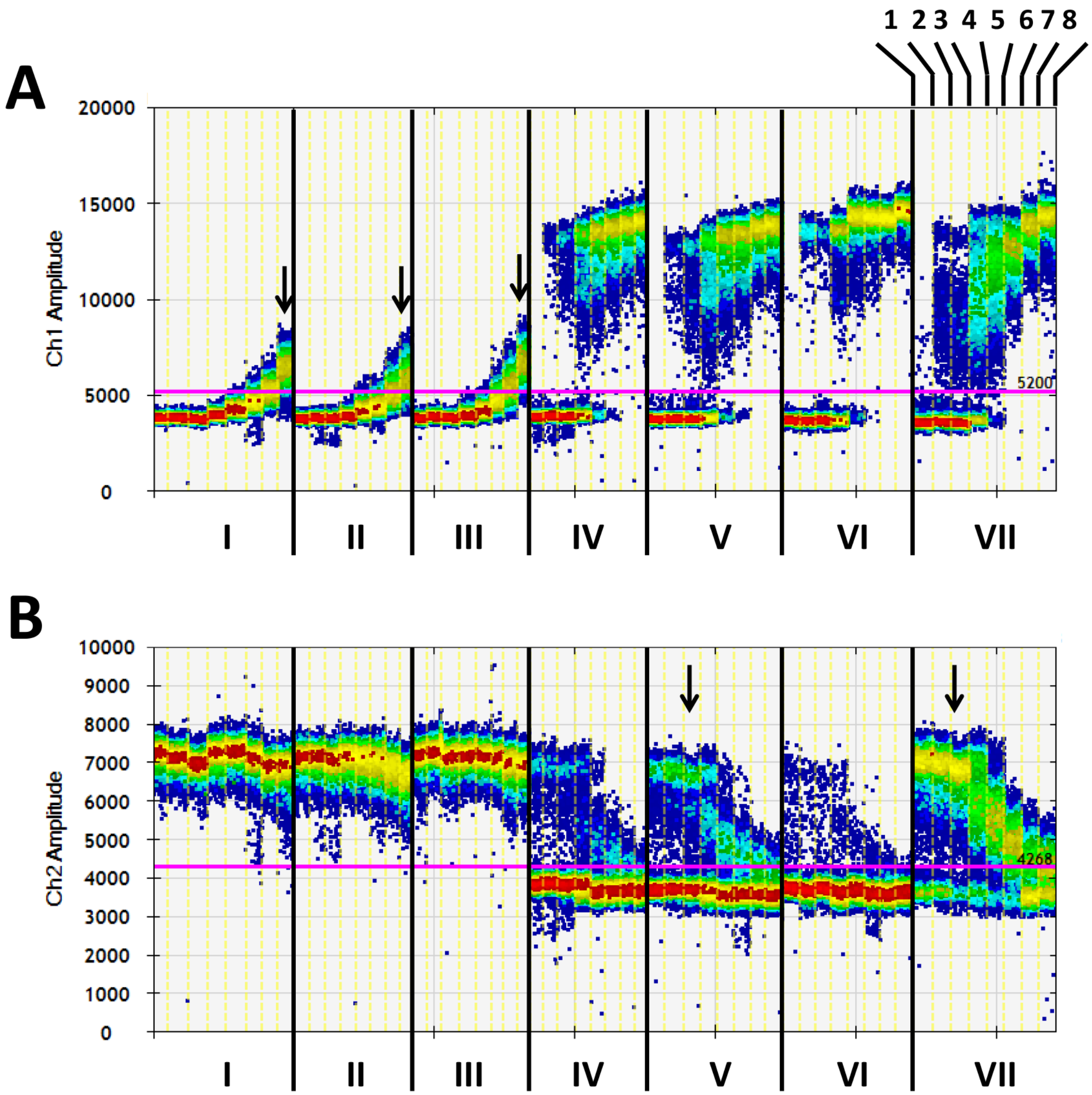

Supplemenary Figure 2: Digital quantification of fluorescence signals was done in standard samples without or with rare number of methylated DNA fragments (0 (1), 5 (2), 9 (3), 94 (4), 375 (5), 750 (6), 1.500 (7) or 3.000 (8) copies as shown at the top on the right side) against a background of unmethylated DNA fragments (25.000 copies) of the *PLA2R1* gene after pre-amplification at 63.0°C for 15 cycles using PL-168 bp, PL-161 bp or PL-150 bp primers. Pre-amplification was followed by ddPCR with the same primer pairs as applied in the pr-eamplification, respectively. **(A)** FAM signals (positive for methylated DNA fragments) registered after pre-amplification with PL-168 bp at an annealing temperature of 55.1°C and 2.5 mM $MgCl_2$ concentration (I), 55.1°C and 8.0 mM $MgCl_2$ (II), 63.0°C and 2.5 mM $MgCl_2$ (III), pre-amplification with PL-150bp at 63.0°C and 2.5 mM $MgCl_2$ (IV), 63.0°C and 8.0 mM $MgCl_2$ (V), or pre-amplification with PL-133bp at 55.1°C and 1.5 mM $MgCl_2$ (VI), and 55.1°C and 6.0 mM $MgCl_2$ (VII). Arrows (in A) show the FAM signal amplitudes in samples with 3,000 copies of methylated DNA using the PL-168bp primer pair (I-III) that were kept low independent on $MgCl_2$ concentration and annealing temperature in comparison to the amplitudes observed while using PL-150 bp and PL-133 bp primer pairs. **(B)** HEX signals (Ch2 amplitude, positive for unmethylated DNA fragments) were registered in the same samples as described in (A). Arrows (in B) show the HEX-positive signal strength using PL-150 bp and PL-133 bp primer pairs dependent on increased $MgCl_2$ concentrations. Fluorescence signals in (A) and (B) are shown as heat map. The used thresholds are shown in red. Results are representative data from three independent experiments.

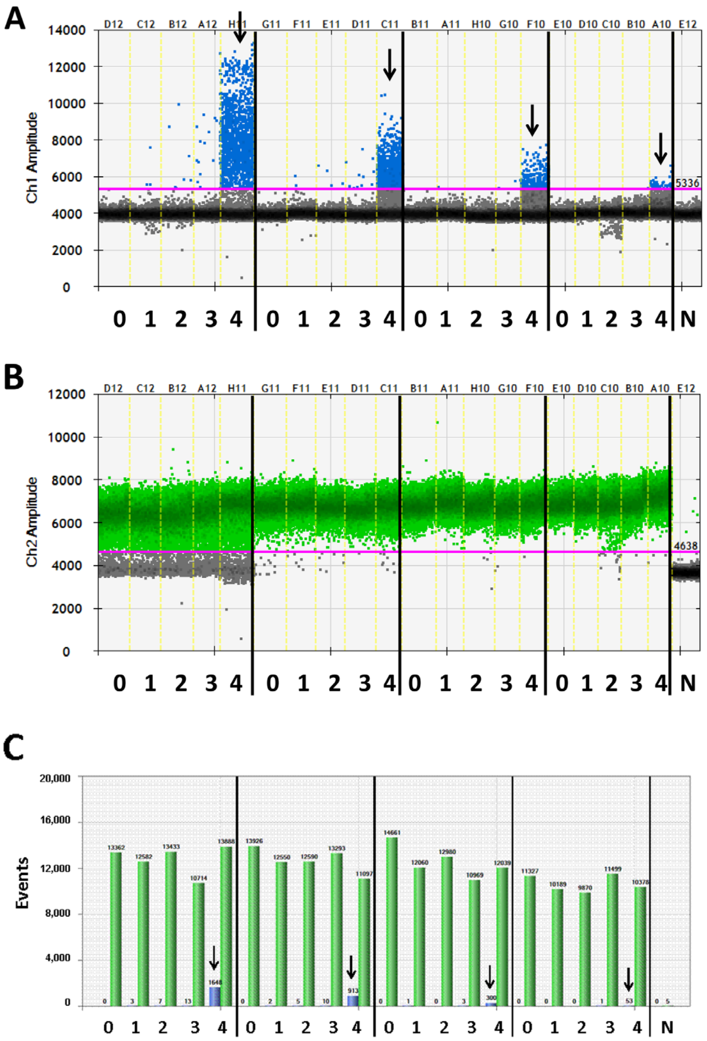

Supplemenary Figure 3: Digital quantification of fluorescence signals was done in samples with rare number of methylated DNA against high background of unmethylated DNA fragments of the *PLA2R1* gene using ddPCR alone (without pre-amplification) with PL-168 bp primer pair. (A) FAM-signals (Ch1 amplitude, positive for methylated DNA fragments in blue) were registered in samples with 70,000 (left from the first black line), 175,000 (left from the second black line), 350,000 (left from the third black line), and 700,000 (left from the fourth black line) copies of unmethylated DNA fragments; without (0) or with 5 (1), 10 (2), 20 (3), and 3,000 (4) copies of methylated DNA fragments. N is the mark for the nontemplate control. Arrows show the signal amplitude in samples with 3.000 copies of methylated DNA fragments. **(B)** HEX signals (Ch2 amplitude, positive for unmethylated DNA fragments in green) were registered in the same samples as described in (A). **(C)** FAM (blue) and HEX (green) positive events were registered in the samples as described in (A and B). Arrows show levels of FAM-positive events in samples with 3,000 copies of methylated DNA. The used thresholds in A and B are shown in red. Raw data for this analysis are summarized in Supplementary Table 1. Results are representative graphs from three independent experiments.

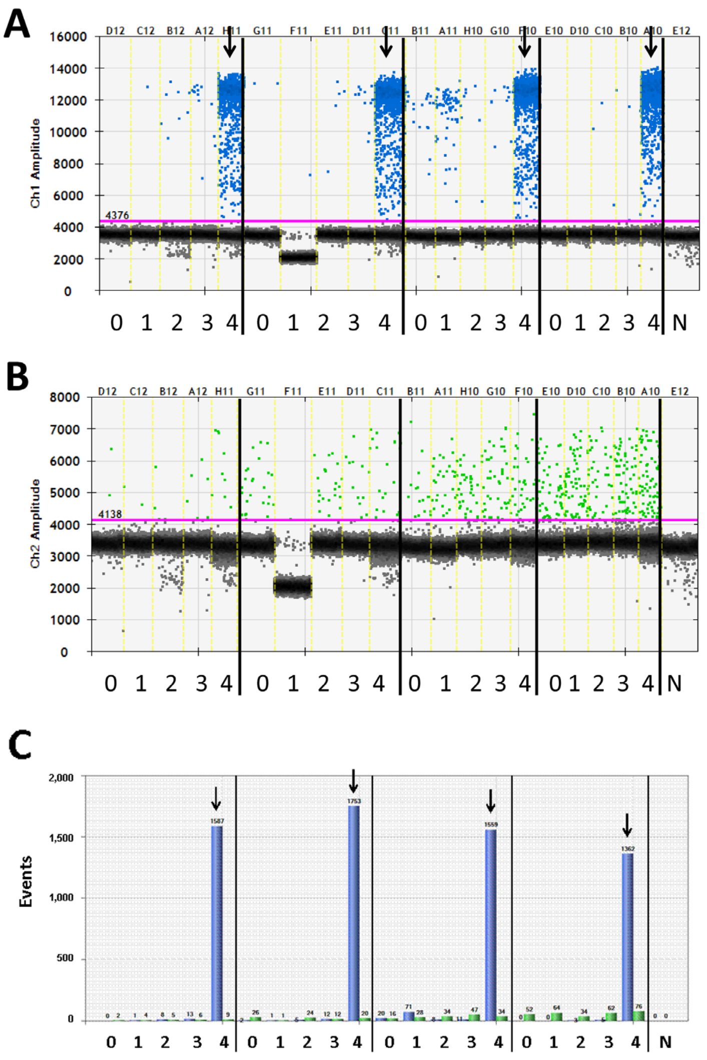

Supplemenary Figure 4: Digital quantification of fluorescence signals was done in samples with rare number of methylated DNA against a high background of unmethylated DNA fragments of the *PLA2R1* gene using ddPCR alone (without pre-amplification) with PL-133 bp primer pair. (A) FAM-signals (positive for methylated DNA fragments in blue) were registered in samples with 70,000 (left from the first black line), 175,000 (left from the second black line), 300,000 (left from the third black line), and 700,000 (left from the fourth black line) copies of unmethylated DNA fragments; and without (0) or with 5 (1), 10 (2), 20 (3), and 3,000 (4) copies of methylated DNA fragments. N is the mark for nontemplate control. Arrows show the signal amplitude in samples with 3,000 copies of methylated DNA fragments. **(B)** HEX signals (positive for unmethylated DNA fragments in green) were registered in the same samples as described in (A). **(C)** FAM (blue) and HEX (green) positive events were registered in the samples described in (A and B). Arrows show levels of FAM-positive events in samples with 3,000 copies of methylated DNA. The used thresholds in A and B are shown in red. Raw data for this analysis are summarized in Supplementary Table 2. Results are representative data from three independent experiments.

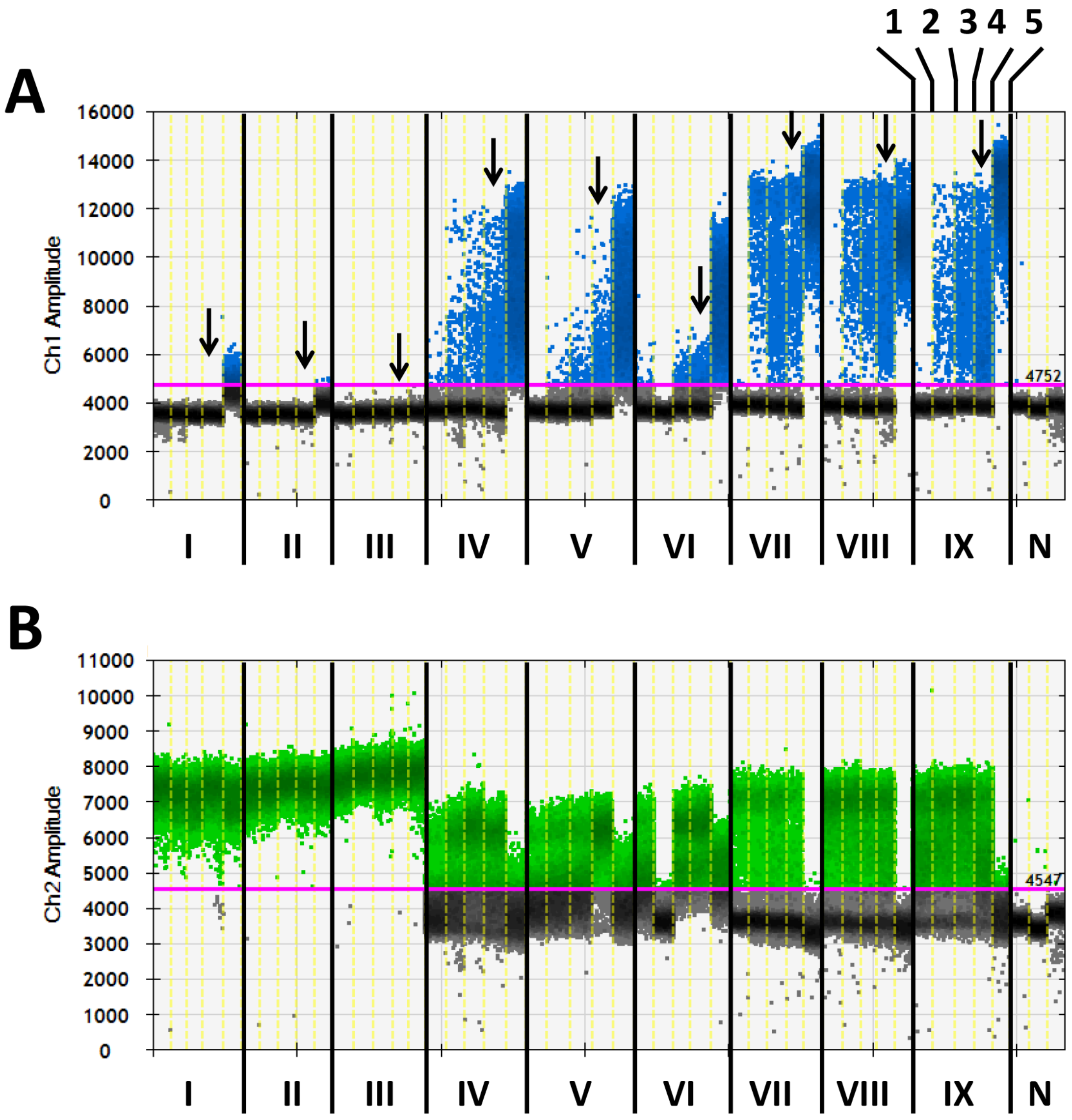

Supplementary Figure 5: Digital quantification of fluorescence signals was done in standard samples with rare number of methylated DNA fragments against a high background of unmethylated DNA fragments of the *PLA2R1* gene using 15 cycles of pre-amplification at 63.0°C and 2.5 mM $MgCl_2$ with PL-168 bp, PL-161 bp, and PL-150 bp primer pairs followed by ddPCR with the same primers. (A) FAM-signals (Ch1 amplitude, positive for methylated DNA fragments in blue) were registered in samples with 0 (1), 5 (2), 10 (3), 20 (4), and 3.000 (5) copies of methylated DNA fragments (as shown at the top on the right side); and 70.000 (I, IV, and VII), 175.000 (II, V, and VIII), and 700.000 (III, VI, and IX) copies of unmethylated DNA fragments using the primer pairs PL-168 bp (I-III), PL-161 bp (IV-VI), and PL-150 bp (VII-IX). N is the mark for nontemplate controls. Arrows show the FAM signal amplitudes in the samples with 20 copies of methylated DNA fragments. **(B)** HEX signals (Ch2 amplitude, positive for unmethylated DNA fragments in green) were registered in the same samples as described in (A). The used thresholds are shown in red. Results are representative data from three independent experiments.

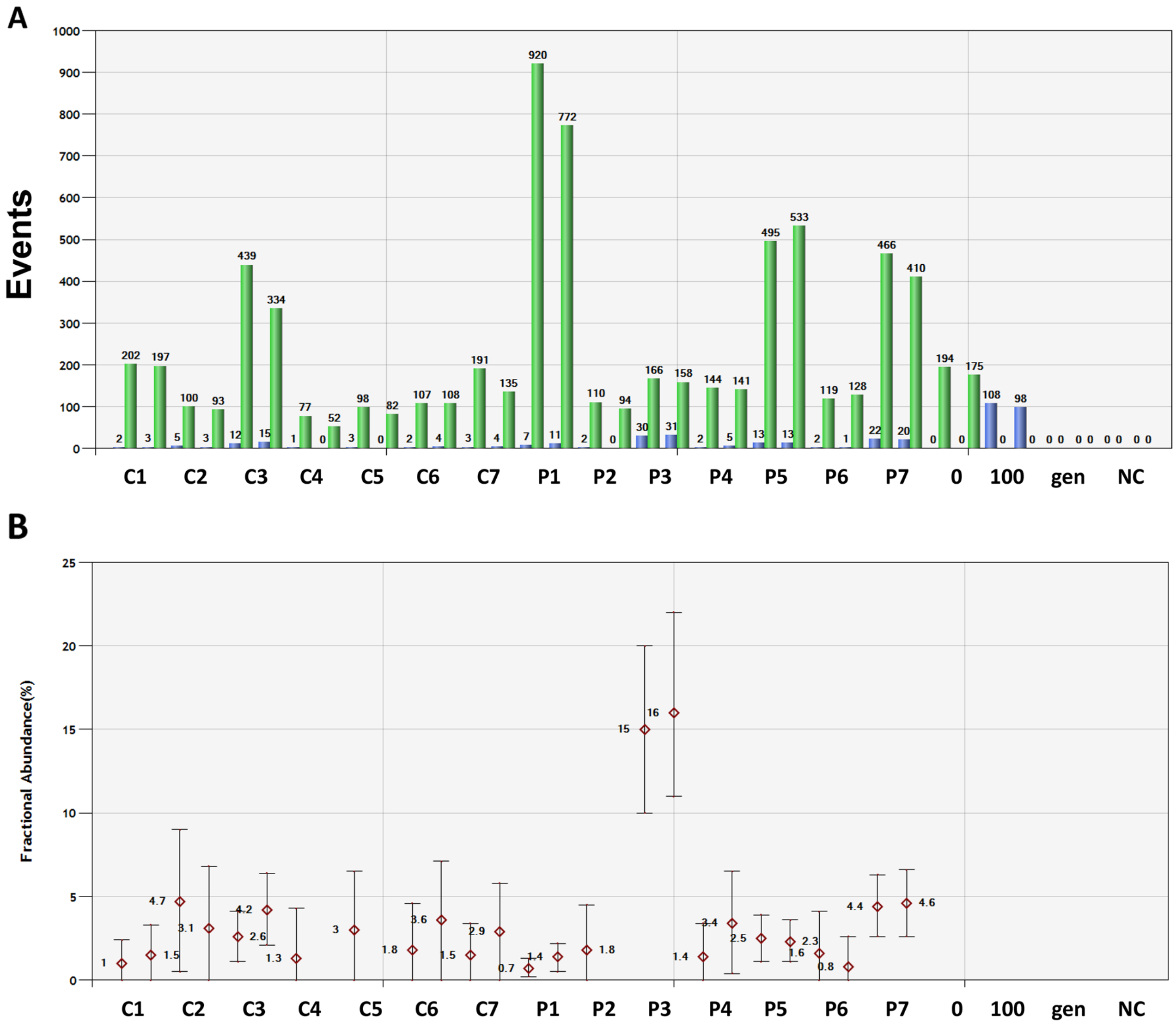

Supplementary Figure 6: **(A)** FAM (blue) and HEX (green) positive events registered through ddPCR alone (without prior pre-amplification) in cfDNA isolated from pooled serum of healthy individuals (C1-C7) and patients with prostate cancer (P1-P7). 0 and 100, standard DNA with methylation degrees of 0% and 100%; gen, genomic DNA without bisulfite modification; NC, non-template control for ddPCR. Results of duplicates are shown and are representative of two independent experiments. **(B)** Fractional abundances [%] and Poisson errors [1] are shown in the same samples as described in Supplementary Figure 6A.

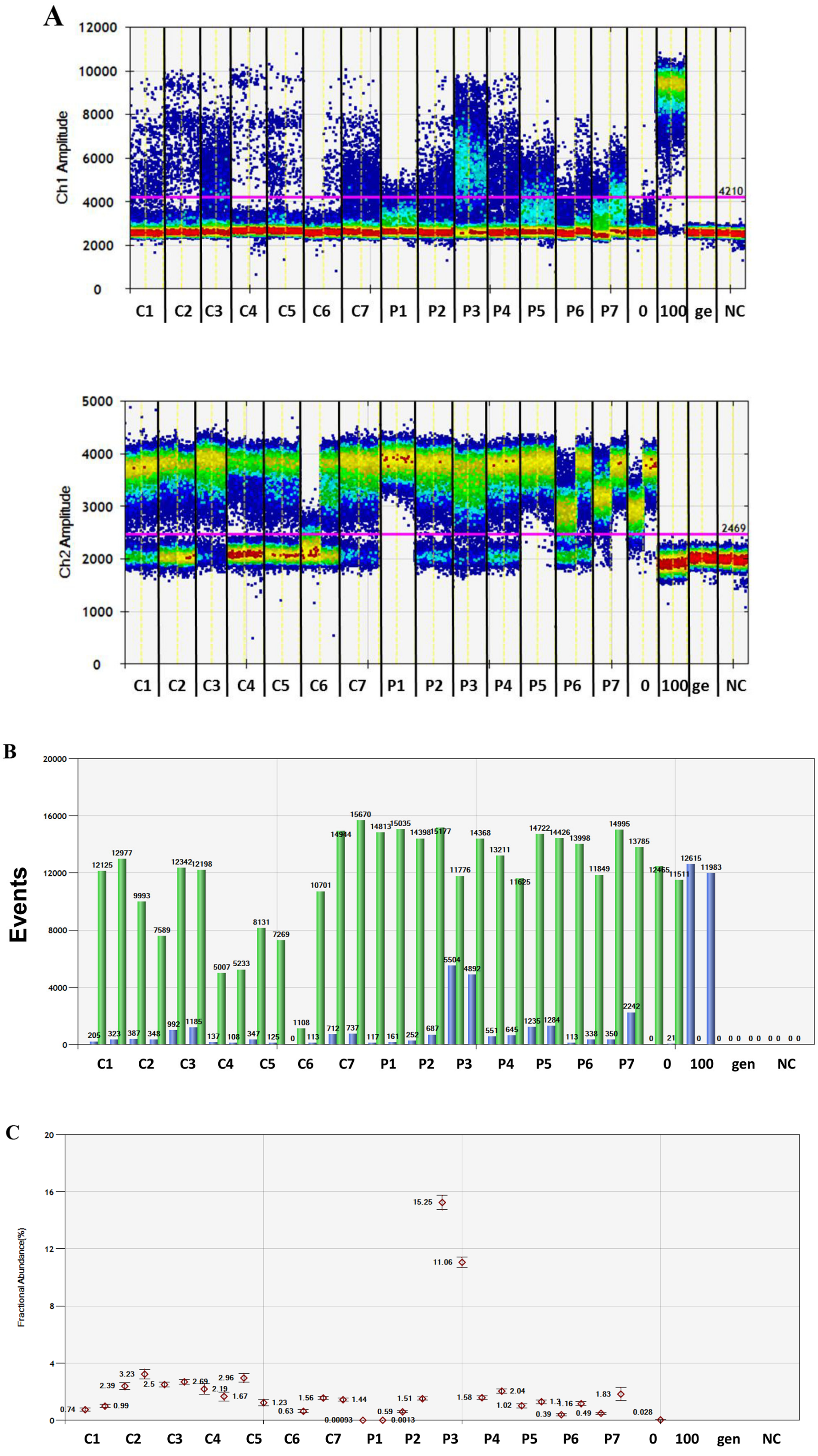

Supplementary Figure 7: (A) Digital quantification of FAM (Ch1 amplitude, positive for methylated DNA fragments in blue) and HEX (Ch2 amplitude, positive for unmethylated DNA fragments in green as heat map) signals in cfDNA isolated from pooled serum samples of healthy individuals (C1-C7) and patients with prostate cancer (P1-P7) after pre-amplification using PL-168bp primer pair with 2.5 mM $MgCl_2$ concentration and at 63.0°C followed by ddPCR. 0 and 100, standard DNA with methylation degrees of 0% and 100%; gen, genomic DNA without bisulfite modification; NC, non-template control for both pre-amplification and ddPCR. Results of duplicates are shown and are representative of two independent experiments. **(B)** FAM (blue) and HEX (green) positive events registered through ddPCR after pre-amplification as described in Supplementary Figure 7A. **(C)** Fractional abundances [%] and Poisson errors [1] are shown in the same samples as described in Supplementary Figure 7A.

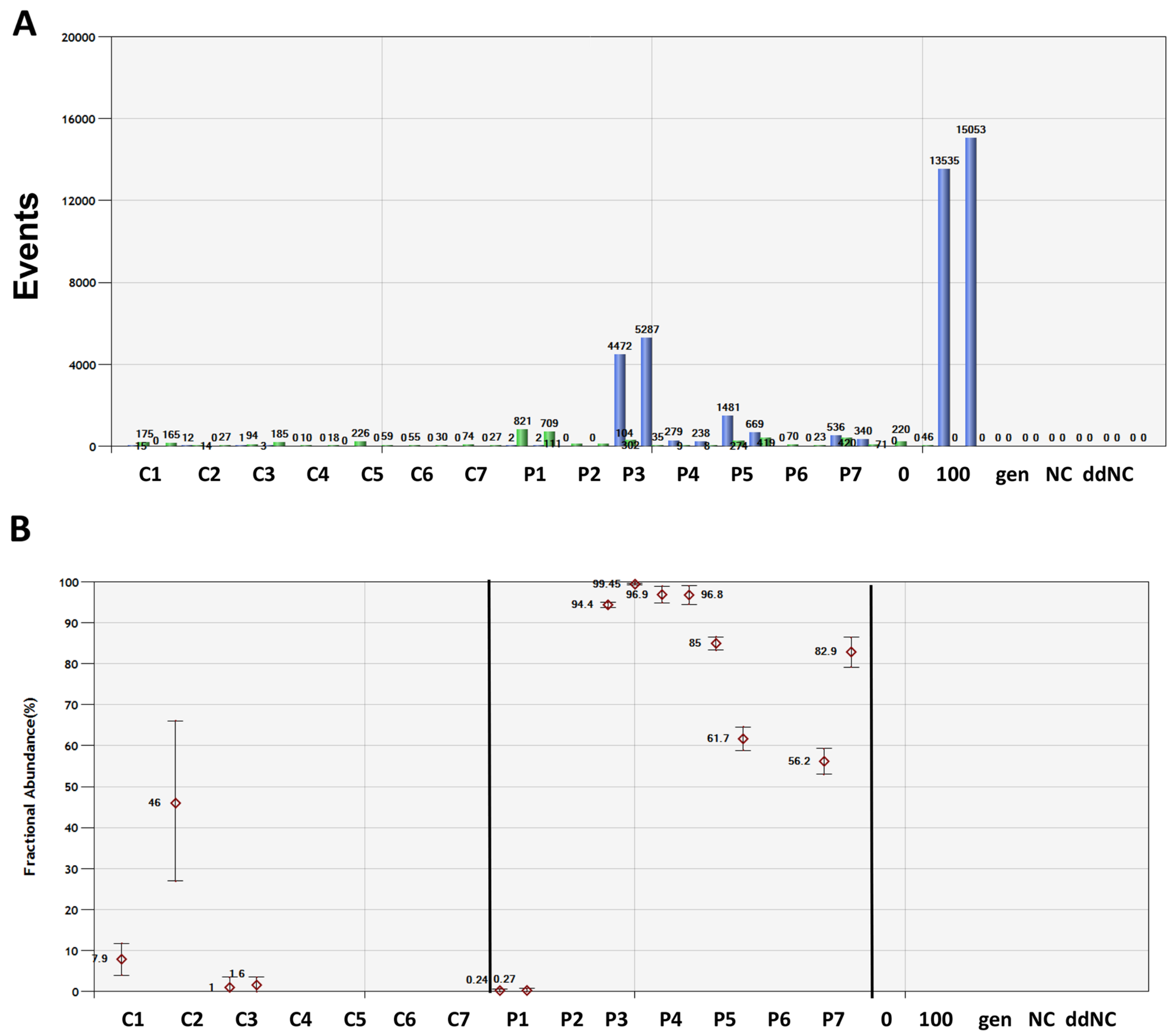

Supplementary Figure 8: **(A)** FAM (blue) and HEX (green) positive events registered through the two-step method consisting in pre-amplification using PL-150bp with 3.5 mM $MgCl_2$ concentration and at 60.8°C followed by ddPCR in cfDNA samples isolated from pooled serum of healthy individuals (C1-C7) and patients with prostate cancer (P1-P7). 0 and 100, standard DNA with methylation degrees of 0% and 100%; gen, genomic DNA without bisulfite modification; NC, non-template control for both pre-amplification and ddPCR and ddNC, non-template control for ddPCR. Results of duplicates are shown and are representative of two independent experiments. **(B)** Fractional abundances [%] and Poisson errors [1] are shown in the same samples as described in Supplementary Figure 8A.

Supplementary Table 1: Copies of methylated (M) and unmethylated (U) *PLA2R1* DNA fragments and resulting fractional abundances after ddPCR alone using MIP PL-168bp primer pair are shown.

See Supplementary File 1

Supplementary Table 2: Copies of methylated (M) and unmethylated (U) *PLA2R1* DNA fragments and resulting fractional abundances after ddPCR alone using MSP PL-133bp primer pair are shown.

See Supplementary File 2
