# Supplementary material for: Identification of rare levels of methylated tumor DNA fragments using an optimized bias based pre-amplification-digital droplet PCR (OBBPA-ddPCR)
Source: Oncotarget. 2018 Nov 16;9(90):36137–50. doi: 10.18632/oncotarget.26315 (PMC6281424; doi:10.18632/oncotarget.26315)
Supplement: Supplementary file 2 [file oncotarget-09-36137-s002.docx]

**Supplementary Table 1:** Copies of methylated (M) and unmethylated (U) *PLA2R1* DNA fragments and resulting fractional abundances after ddPCR alone using MIP PL-168bp primer pair are shown. Sample 0/70K (no copies of methylated DNA and 70,000 unmethylated DNA), 5/70K (5 copies of methylated DNA and 70,000 unmethylated DNA), 10/70K (10 copies of methylated DNA and 70,000 unmethylated DNA), 20/70K (20 copies of methylated DNA and 70,000 unmethylated DNA) and 3,000/70K (3,000 copies of methylated DNA and 70,000 unmethylated DNA). 0/175K-3,000/175K, 0/350-3,000/350K, and 0/700K-3,000/700K indicate samples with 0, 5, 10, 20 and 3,000 copies of methylated DNA in backgrounds of 175,000, 350,000 and 700,000 copies of unmethylated DNA, respectively. M, probe specific for methylated DNA fragments; U, probe specific for unmethylated DNA fragments.

| Samples | probes | copies/  20 µl well | Positives | Negatives | Ch1+Ch2+ | Ch1+Ch2- | Ch1-Ch2+ | Ch1-Ch2- | accepted  droplets | ratio | fractional  abundance |
| --- | --- | --- | --- | --- | --- | --- | --- | --- | --- | --- | --- |
| 0/70K | M | 0 | 0 | 14071 | 0 | 0 | 13362 | 709 | 14071 | 0 | 0 |
|  | U | 70400 | 13362 | 709 |  |  |  |  |  |  |  |
| 5/70K | M | 5.4 | 3 | 13198 | 3 | 0 | 12579 | 619 | 13201 | 7,00E-05 | 0.007 |
|  | U | 72000 | 12582 | 619 |  |  |  |  |  |  |  |
| 10/70K | M | 11.6 | 7 | 14085 | 5 | 2 | 13428 | 657 | 14092 | 0.00016 | 0.016 |
|  | U | 72000 | 13433 | 659 |  |  |  |  |  |  |  |
| 20/70K | M | 28 | 13 | 11255 | 12 | 1 | 10702 | 553 | 11268 | 0.00038 | 0.038 |
|  | U | 70800 | 10714 | 554 |  |  |  |  |  |  |  |
| 3.000/70K | M | 2780 | 1648 | 13095 | 1483 | 165 | 12405 | 690 | 14743 | 0.0416 | 4 |
|  | U | 67000 | 13888 | 855 |  |  |  |  |  |  |  |
| 0/175K | M | 0 | 0 | 13938 | 0 | 0 | 13926 | 12 | 13938 | 0 | 0 |
|  | U | 166000 | 13926 | 12 |  |  |  |  |  |  |  |
| 5/175K | M | 3.8 | 2 | 12554 | 2 | 0 | 12548 | 6 | 12556 | 2.1E-05 | 0.0021 |
|  | U | 180000 | 12550 | 6 |  |  |  |  |  |  |  |
| 10/175K | M | 9.4 | 5 | 12592 | 5 | 0 | 12585 | 7 | 12597 | 5.3E-05 | 0.0053 |
|  | U | 176000 | 12590 | 7 |  |  |  |  |  |  |  |
| 20/175K | M | 17.8 | 10 | 13286 | 10 | 0 | 13283 | 3 | 13296 | 9,00E-05 | 0.009 |
|  | U | 198000 | 13293 | 3 |  |  |  |  |  |  |  |
| 3.000/175K | M | 2018 | 913 | 10193 | 909 | 4 | 10188 | 5 | 11106 | 0.0121 | 1.19 |
|  | U | 168000 | 11097 | 9 |  |  |  |  |  |  |  |
| 0/350K | M | 0 | 0 | 14661 | 0 | 0 | 14661 | 0 | 14661 | 0 | 0 |
|  | U | 20000000 | 14661 | 0 |  |  |  |  |  |  |  |
| 5/350K | M | 2 | 1 | 12059 | 1 | 0 | 12059 | 0 | 12060 | 1,00E-07 | 1,00E-05 |
|  | U | 20000000 | 12060 | 0 |  |  |  |  |  |  |  |
| 10/350K | M | 0 | 0 | 12984 | 0 | 0 | 12980 | 4 | 12984 | 0 | 0 |
|  | U | 190000 | 12980 | 4 |  |  |  |  |  |  |  |
| 20/175K | M | 6.4 | 3 | 10969 | 3 | 0 | 10966 | 3 | 10972 | 3.3E-05 | 0.0033 |
|  | U | 194000 | 10969 | 3 |  |  |  |  |  |  |  |
| 3.000/175K | M | 594 | 300 | 11740 | 300 | 0 | 11739 | 1 | 12040 | 0.0027 | 0.27 |
|  | U | 222000 | 12039 | 1 |  |  |  |  |  |  |  |
| 0/700K | M | 0 | 0 | 11327 | 0 | 0 | 11327 | 0 | 11327 | 0 | 0 |
|  | U | 20000000 | 11327 | 0 |  |  |  |  |  |  |  |
| 5/700K | M | 0 | 0 | 10189 | 0 | 0 | 10189 | 0 | 10189 | 0 | 0 |
|  | U | 20000000 | 10189 | 0 |  |  |  |  |  |  |  |
| 10/700K | M | 0 | 0 | 9889 | 0 | 0 | 9870 | 19 | 9889 | 0 | 0 |
|  | U | 148000 | 9870 | 19 |  |  |  |  |  |  |  |
| 20/700K | M | 2 | 1 | 11499 | 1 | 0 | 11498 | 1 | 11500 | 9,00E-06 | 0.0009 |
|  | U | 220000 | 11499 | 1 |  |  |  |  |  |  |  |
| 3.000/700K | M | 120 | 53 | 10329 | 53 | 0 | 10325 | 4 | 10382 | 0.00065 | 0.065 |
|  | U | 184000 | 10378 | 4 |  |  |  |  |  |  |  |
| NTC | M | 0 | 0 | 11795 | 0 | 0 | 5 | 11790 | 11795 | 0 | 0 |
|  | U | 10 | 5 | 11790 |  |  |  |  |  |  |  |
